# The Quality of the Herbal Product Obtained in the Pressure Agglomeration Process

**DOI:** 10.3390/ma18040799

**Published:** 2025-02-12

**Authors:** Sadowska Urszula, Żabiński Andrzej, Kukiełka Ewelina, Kopeć Aneta, Mudryk Krzysztof

**Affiliations:** 1 Institute of Machinery Exploitation, Ergonomics and Production Processes, University of Agriculture in Krakow, Łupaszki 6, 30-198 Krakow, Poland; andrzej.zabinski@urk.edu.pl; 2 Department of Human Nutrition, Faculty of Food Technology, University of Agriculture in Krakow, Balicka 122, 30-149 Krakow, Poland; ekukiel@gmail.com (K.E.); aneta.kopec@urk.edu.pl (K.A.); 3 Department of Mechanical Engineering and Agrophysics, University of Agriculture in Krakow, Balicka 120, 30-149 Krakow, Poland; krzysztof.mudryk@urk.edu.pl

**Keywords:** lemon balm, agglomeration process, mechanical properties, antioxidant activity, polyphenols

## Abstract

The aim of the conducted research was to evaluate the impact of the pressure agglomeration process of lemon balm (*Melissa officinalis* L.) on the mechanical properties of the obtained product, its antioxidant capacity, and total polyphenol content. Two fractions of lemon balm were isolated with particle sizes of 0.5–2.5 mm and 2.5–5.0 mm. The isolated fractions were compacted using a Fritz Heckert EU 20 hydraulic press, applying compaction pressures of 100, 150, and 200 MPa. A closed die was used, with 2 g of the plant sample introduced each time. The mechanical properties of the obtained product were determined through an abrasion test and diameter test (Brazilian method) using the MTS Insight 2 testing machine. The total polyphenol content and antioxidant activity were measured using the ABTS method, both directly after product preparation and after a 6-month storage period. The compaction of lemon balm resulted in an increase in total polyphenol content and antioxidant properties compared to the unpressed raw material. The obtained product displayed favorable mechanical properties, as confirmed by the conducted mechanical tests. Regardless of the applied herb fraction, an agglomeration pressure of 200 MPa is particularly recommended.

## 1. Introduction

Lemon balm (*Melissa officinalis* L.) is a medicinal herb belonging to the Lamiaceae family, widely known and used around the world [1]. The largest global producers include Egypt, Turkey, the United Kingdom, and the United States [2]. In Poland, this species is cultivated on approximately 5000 hectares nationwide [3]. Poland is among the largest producers in Europe, with an annual production approaching 1500 tons [4]. Lemon balm and its products are commonly used in Europe, primarily for their calming and antidepressant effects in states of nervous arousal, insomnia, and vegetative neurosis. Additionally, they exhibit antispasmodic and antibacterial properties and stimulate digestion [5,6,7]. Studies on hyperactive children investigating the sedative effects of this species in combination with valerian have shown promising results [8]. Currently, consumers put significant emphasis on food properties, such as smell, taste, and appearance [9]. The introduction of a new product to the market may contribute to health-promoting initiatives. The use of local medicinal herbs is also recommended by the World Health Organization [10]. Nowadays, there is growing interest among scientists, consumers, and food industry manufacturers in food products that, in addition to their traditional nutritional value, offer health-promoting benefits. Herbal infusions are one of the most common beverages in the world. Recently, an upward trend in their sales has been observed in Europe [11].

The pressure agglomeration process is commonly used in the agri-food, chemical, pharmaceutical, ceramic, metallurgical, and other industries [12,13,14]. The main goal of agglomeration is to obtain a product with a minimal dust content. This process, by increasing the density of the raw material, facilitates its storage, transport, and dosing in automated production processes [15]. The physical and chemical properties of raw materials determine their susceptibility to granulation. Good susceptibility means that the material can be compacted with lower energy input, and the resulting agglomerate has appropriate hardness and resistance to crushing and shattering [16,17]. The main parameters indicating the quality of products obtained by pressure agglomeration are primarily their density and mechanical strength [18]. However, it should be emphasized that as the density increases, the brittleness of the granules also increases [19]. The pressure used during agglomeration plays a critical role in the process and the quality of the obtained product [20]. Inappropriate values of this parameter may, on the one hand, lead to useless energy consumption and, on the other hand, result in obtaining an agglomerate with inadequate strength parameters. The densification of plant materials is also used to stabilize certain groups of biologically active substances. However, during the agglomeration process, the material is exposed to various groups of factors, mechanical, thermal, and pressure, which may also negatively affect the content of active ingredients. Volatile components of essential oils found in herbal plants are particularly sensitive to such effects [21].

Recent research results confirm that herbs are a rich source of natural antioxidant compounds [22,23,24]. This is particularly significant due to their potential health-promoting effects and prevention of lifestyle diseases. Among the most important compounds influencing antioxidant activity, as well as affecting the taste and color of food products, are polyphenols [25,26,27]. Phenolic compounds are a group of plant secondary metabolites, and their stability depends on many factors, including temperature, pH, and light or oxygen exposure. Some groups are stable under various thermal treatments. For example, blanching, vacuum treatment, pasteurization, and steaming may increase the content of vanillic acid, cinnamic acid, and 4-hyroxybenzoic acid. Oppositely, conventional cooking may decrease the content of caffeic acid, ferulic acid, and sinapic acid [28,29]. It was reported that the use of high hydrostatic pressure (water at room temperature) generally increases the content of bioactive compounds including phenolic compounds in plant material. This can be explained by the mechanical damage to cell walls and the release of polyphenolic compounds, which can result in improved extraction of polyphenols from plant material. Mechanical damage to the plant material results in increased stress in that material and increased synthesis of polyphenolic compounds [28,30,31]. In the study by Sadowska et al. [32], it was also found that the use of pressure for the preparation of agglomerates of peppermint resulted in increasing the content of total polyphenolic compounds. The aim of the conducted research was to assess the impact of the pressure agglomeration process of lemon balm herb on the mechanical properties, as well as the total polyphenol content and antioxidant capacity, of the obtained product both directly after product preparation and after a 6-month storage period.

## 2. Materials and Methods

### 2.1. Plant Material

The experimental material consisted of naturally dried lemon balm herb obtained from a production plantation. The humidity of the raw material was around 10%, which is the upper pharmaceutically acceptable norm for this species [33]. Using a laboratory shaker LPzE-2e (MULTISERW-Morek, Marcyporęba, Poland) of vibration amplitude 0–2.5 mm, and sieves with appropriate mesh sizes, two fractions of lemon herb were separated: 0.5–2.5 mm and 0.5–5 mm.

### 2.2. Methods

#### 2.2.1. Lemon Balm Herb Pressure Agglomeration Process

**Figure 1:**
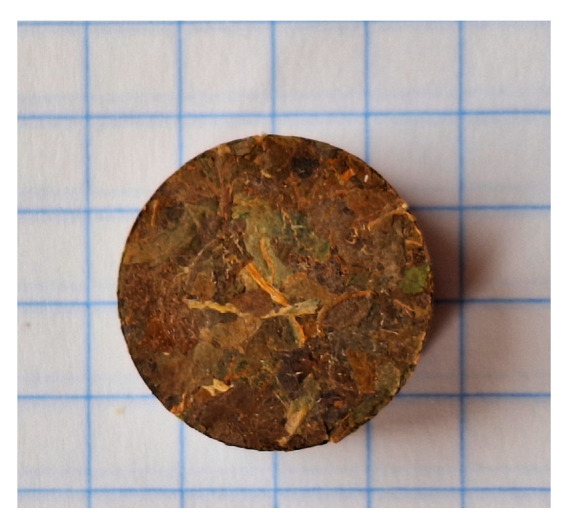
An example of agglomerate appearance formed under the pressure of 200 MPa.

The prepared lemon balm herb fractions were then subjected to agglomeration via a hydraulic testing machine (Fritz Heckert EU 20, Karl-Marx-Stadt, Germany), utilizing the following compaction pressures: 100, 150, and 200 MPa. A closed die with a pressing chamber diameter of 15.6 mm was used. The herb samples, with a mass of 2 g, were manually introduced into the working chamber, and then compaction was carried out under pressure control. The resulting samples were found to be cylindrical in shape, with a consistent diameter (see Figure 1). Following the compaction process, the samples were subjected to a stabilization process for 48 h to ensure the stability of the internal stresses.

#### 2.2.2. Strength Testing of Granules

The mechanical characterization of the granular samples obtained was carried out by conducting abrasion tests and static compressive strength tests.

**Figure 2:**
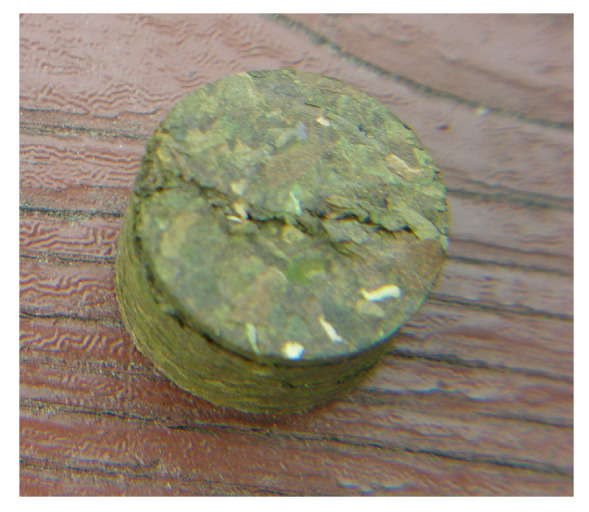
An example of agglomerate appearance obtained in the Brazilian test.

(1) The abrasion test of the granules was conducted in accordance with the established methodology for evaluating uncoated tablets intended for pharmaceutical use, as delineated in the European Pharmacopoeia [33]. A comprehensive description of the procedure was provided in the publication by Sadowska and colleagues [32], in which the abrasion strength of peppermint granules was assessed. The experimental design entailed two sets of abrasion tests: the first set was conducted immediately following the agglomeration process, which was followed by a stabilization period, and the second set was performed after a three-month storage period. The primary objective of the latter test was to assess whether the storage period exerted an influence on the mechanical properties of the granules.
(2) The determination of the static strength of the obtained granules was carried out by performing radial compression (Brazilian test) 48 h after the agglomerate was produced and again after a storage period of 3 months. In this test, the cylindrical sample was subjected to compressive loading along its diameter. This type of loading causes the sample to fail when the tensile strength, in the direction perpendicular to the plane of symmetry of the granule containing the load direction, is exceeded. The tests were conducted using an Insight 2 strength testing machine (MTS, Eden Prairie, MN, USA), where the loading process was set under quasi-static conditions. The compaction process was continued until fractures originating in the central part of the sample (cylinder) were visible to the naked eye (see Figure 2).

The tensile strength under radial compression was calculated based on the following relationship:

(1) $$\delta =2\cdot Fn\cdot {\left(\pi \cdot d\cdot l\right)}^{-1}$$

δ—tensile strength under radial compression (Pa);
*Fn*—force destroying the agglomerate (N);
*d*—diameter of the compacted sample (m);
*l*—length of the agglomerate cylinder (m).

In order to provide comprehensive physical information about the analyzed samples, the geometry of the granules was measured to determine the specific density (kg·m^−3^). Geometric measurements were made with a caliper (MAUa 150, VIS S.A, Warsaw, Poland ±0.03 mm) to determine the diameter and length of 20 randomly selected granules for each test variant, while measurements of the mass of granules were made in the container with five repetitions using a laboratory balance WPS 360/C/1 (RADWAG, Radom, Poland) with an accuracy of ±0.001 g.

#### 2.2.3. Extracts Preparation

To measure the total polyphenol content and antioxidant activity, 0.3 g of lemon balm sample was weighed into an Erlenmeyer flask. An amount of 80 mL of 70% methanol (POCH, Gliwice, Poland) was added. The mixtures were extracted at room temperature for 2 h by shaking without light (Elpan, shaker type 357, Lubawa, Poland). Samples were then centrifuged at 1500 rpm for 15 min (Centrifuge type MPW-340, Warsaw, Poland). The supernatants were decanted and stored at temperature −20 °C until analysis.

#### 2.2.4. Determination of Total Polyphenols

The content of total polyphenols extracts was estimated using the Folin–Ciocalteu reagent (Sigma-Aldrich, St. Louis, MO, USA) according to the method of Swain and Hillis [34]. Extracts were diluted at a ratio of 1:20 with distilled water. Reaction mixtures were prepared by mixing 5 mL of diluted extract, 0.5 mL of Folin–Ciocalteu reagent, and 0.25 mL of 25% sodium carbonate (POCH, Gliwice, Poland). The samples were allowed to stand for 20 min. Absorbance at 760 nm was measured using a spectrophotometer (UV-1800, RayLeigh, Beijing Beifen-Ruili Analytical Instrument Co., Ltd., Beijing, China). The results were expressed as chlorogenic acid equivalents (CGA) in mg CGA per 100 g sample.

#### 2.2.5. Determination of Antioxidant Activity

The extracts were used to determine (spectrometrically) the antioxidant activity by identifying the ability of the sample to quench an ABTS-+ (2,2′-azinobis-(3-ethylbenzothiazoline-6-sulphonic acid) free radical [35]. The method involved the colorimetric determination of the amount of colored solution of ABTS-+ reduced by the antioxidants present in the product analyzed. Absorbance was measured at a wavelength of 734 nm using a spectrometer (UV-1800, Ray-Leigh, Beijing Beifen-Ruili Analitical Instrument Co., Ltd., Beijing, China). The values obtained for each sample were compared with the concentration–response curve of the Trolox standard solution and expressed as micromoles of Trolox equivalent per gram of fresh weight (TEAC).

#### 2.2.6. Statistical Analysis

The results were analyzed with the use of STATISTICA v.13.3 (StatSoft Inc., Tulsa, OK, USA). Two-way factorial analysis of variance (MANOVA) was used to test the differences. The significance of the obtained differences was verified with a Duncan test at the significance level of *p* ≤ 0.05. The results of the statistical evaluation are presented in tables, together with the identification of homogeneous groups marked using letter classification. The correlation between variables was assessed using the Pearson linear correlation coefficient (r), with a significance level of *p* ≤ 0.05.

## 3. Results

### 3.1. Strength Tests

One of the fundamental criteria for evaluating the quality of an agglomerate is its mechanical strength. This attribute signifies the capacity of the product to sustain its structural integrity under specific adverse physical external conditions. This characteristic is of paramount importance not only in the utilization of the finished product but also during its distribution, associated with storage and transportation.

The values obtained from the abrasion tests conducted immediately after the production of the agglomerate and after a three-month storage period are presented in Table 1 and Table 2, respectively. The findings revealed a substantial impact of the applied pressure on agglomerate resistance to abrasion. It was observed that an increase in pressure led to a greater reduction in weight loss of the agglomerate, a phenomenon observed in both tests. However, the observed weight loss was less than 1% in all cases, which is the maximum acceptable limit for uncoated tablets as defined by the European Pharmacopeia [33]. Directly following product formation, agglomerates derived from the 2.5–5.0 mm fraction exhibited reduced abrasiveness; however, these disparities became indistinguishable after a three-month storage period. A relationship was also observed between the applied pressure and the separated fraction of lemon balm herb. Immediately following the agglomeration process, the smallest mass losses were found at the higher pressures of 150 and 200 MPa for the 2.5–5.0 mm fraction and the pressure of 200 MPa for the 0.5–2.5 mm fraction. Subsequent to the initial testing period, a second testing period was conducted after three months for both fractions. The lowest losses were observed for the agglomerate produced at 200 MPa pressure.

To adequately describe the alterations in the mechanical properties of the obtained granules in response to the applied agglomeration pressures, a Brazilian test, also known as a diameter test, was conducted (see Table 3).

**Figure 3:**
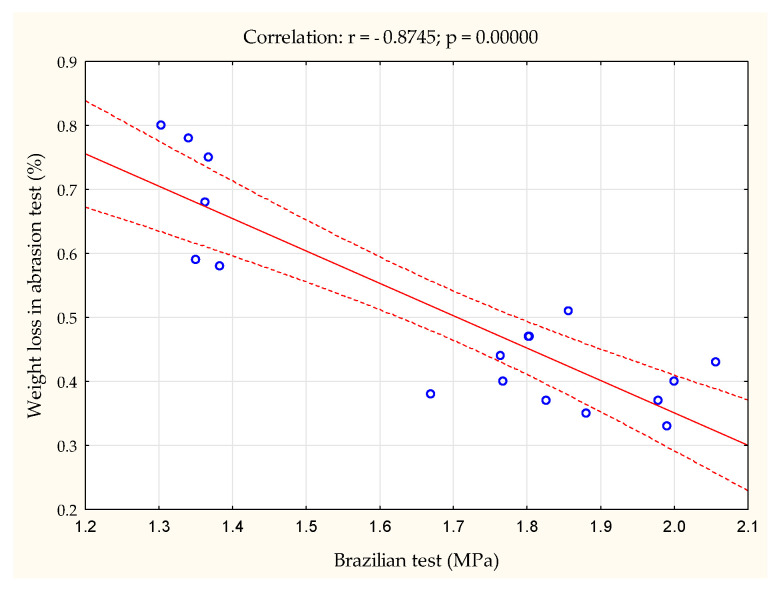
Relationship between agglomerate abrasion test results and Brazilian (diametral) test.

The highest strength in the diameter test (load applied along the diameter) was exhibited by granules formed from the 0.5–2.5 fraction at a pressure of 200 MPa, while the lowest strength was exhibited by granules formed at a pressure of 100 MPa, regardless of the raw material fraction. In summary, the strength of the agglomerate produced from lemon balm herb with particles in the 0.5–2.5 mm range was higher compared to that obtained from the 2.5–5.0 raw material fraction, but only for pressures of 150 and 200 MPa. The differences in strength were minimal, amounting to 0.092 and 0.11 MPa, respectively. The observed correlations in the abrasiveness of the produced agglomerate were also confirmed by the obtained values of the Brazilian test, and a high negative correlation between these tests was found (see Figure 3). Generalizing, it can be said that an increase in the value causing agglomerate destruction may indicate the agglomerate’s greater resistance to abrasion. Thus, the results of the Brazilian test can, to some extent, reflect trends related to the agglomerate’s susceptibility to abrasion.

Another parameter indicating the quality of products obtained by pressure agglomeration is their density. The density of granules obtained from lemon balm herb ranged from 1018.891 to 1141.285 kg·m^−3^. It has been demonstrated that an augmentation in agglomeration pressure results in an increase in granule density, a phenomenon observed across both fractions of the herb utilized. Specifically, the specific density of the agglomerate produced from the finer fraction of the raw material at pressures of 150 and 200 MPa exhibited higher values compared to the fraction with larger particles for each of the aforementioned pressures (see Table 4).

Concurrent findings on the density of pellets made from dried parsley and chives were reported by Mudryk et al. [36], with a range of specific densities from 1012.7 to 1104.3 kg·m^−3^. The pellets in this study were produced on a semi-industrial pelletizer with a diameter of compaction channels equal to 8 mm.

**Figure 4:**
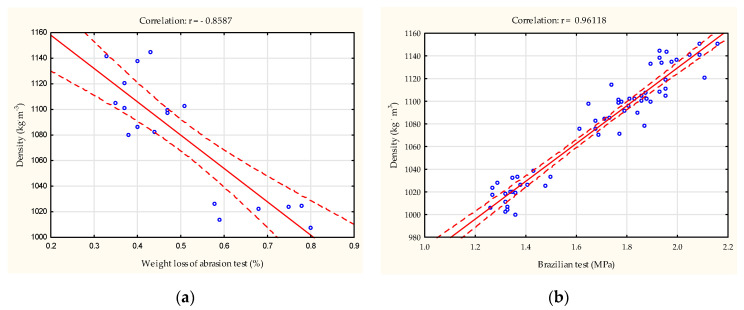
The correlation between the product density and the abrasion test (**a**) and the Brazilian test (**b**) was examined.

The correlation relationships between the strength tests utilized in the study and the density of the resulting product are demonstrated in Figure 4a,b. An enhancement in agglomerate density led to a reduction in agglomerate weight loss during the abrasion test. Additionally, an enhancement in agglomerate strength parameters, as measured by the Brazilian test, was observed, exhibiting a high correlation coefficient of r = 0.96 with an increase in density.

### 3.2. Antioxidant Properties and Total Polyphenol Content

The results of the studies on total polyphenol content and antioxidant properties measured by the ABTS test, performed directly after agglomerate production, are presented in Table 5, while those after the storage period are presented in Table 6.

The pressure agglomeration of lemon balm, regardless of the applied pressure value and the herb fraction, resulted in an increase in the total polyphenol content compared to the loose herb. Immediately after agglomerate formation, the highest polyphenol content was observed in the agglomerate produced at 100 MPa from the 0.5–2.5 mm fraction (3762.81 mg·100 g⁻¹). Similarly, for the thicker herb fraction (2.5–5.0 mm), the highest polyphenol concentration was found in the agglomerate produced at 100 MPa (2951.92 mg·100 g⁻¹). After storage, the most abundant in polyphenols among all samples was the agglomerate produced from the coarser fraction under pressures of 100 and 150 MPa, 1592.30 and 1500.93 mg·100 g^−1^, respectively. Similarly, the highest amount of polyphenols in the finer fraction of the compressed lemon balm herb was achieved at pressures of 100 and 150 MPa, 1456.80 and 1472.27 mg·100 g^−1^, respectively. Analogously to the total polyphenol content, significantly higher antioxidant activity as measured by the ABTS test was observed in the compressed lemon balm herb compared to the loose one. Immediately after the agglomerate production, the most favorable results were achieved for the 2.5–5.0 mm fraction at 100 MPa and the 0.5–2.5 mm fraction and 200 MPa pressure, which were 175.25 and 170.06 μmol Trolox·g^−1^, respectively. After the storage period, no more significant differences in antioxidant activity were observed between the applied agglomeration pressures. However, similar to the results immediately after production, the lowest antioxidant activity was found in the loose lemon balm.

## 4. Discussion

One of the basic criteria for evaluating the quality of an agglomerate is its mechanical strength. This parameter serves as an indicator of the product’s ability to maintain its structural integrity under specific adverse conditions involving external physical factors. This property is of critical importance not only in the utilization of the final product but also during its distribution, which involves storage and transportation. The results of studies on the mechanical strength of the produced agglomerate, measured using both the abrasion test and the Brazilian test, indicate a significant influence of the applied pressure on this parameter. As the applied pressure increased within the range of 100, 150, and 200 MPa, the abrasion test revealed a reduced mass loss of the produced agglomerate and an increase in the force required to cause its fracture. Furthermore, it was observed that the agglomerate’s density also increased with the applied pressure. The correlation relationships between the results obtained in the various strength tests and the density of the agglomerate were high, indicating the influence of this parameter on the mechanical strength of the obtained product. Similarly, Stelte et al. [37] indicate that as the applied pressure increases, the density of pellets made from plant materials increases. Consequently, it is expected that the product obtained at higher pressure will have the highest degree of agglomerate density and thus more favorable mechanical performance. Similar results during the pressure agglomeration of peppermint herb were achieved by Sadowska et al. [32]. In the cited studies, a lower weight loss of agglomerate with increasing pressure was also observed. In the results of pressure agglomeration studies of lemon balm presented in this paper, a smaller weight loss of agglomerate produced from the coarser fraction of the herb was noted. However, this phenomenon was visible only immediately after its production. The storage period canceled out these differences. This phenomenon is also highlighted in the study by Sadowska et al. [32]. The density of agglomerates produced from finer raw material fractions under pressures of 150 and 200 MPa was higher compared to those made from larger particle fractions at each of the pressures mentioned above (Table 4). The influence of material fineness on agglomerate density is corroborated by research conducted by Carone et al. [38] on the compaction of olive tree waste. Their findings indicate that the density of the resulting agglomerate increases as the particle size of the raw material decreases.

This trend was further confirmed by the mechanical strength results obtained in the diameter (Brazilian) test. The strength of agglomerates produced from plant material with particle sizes ranging from 0.5 to 2.5 mm was higher than that of agglomerates made from raw material fractions of 2.5 to 5.0 mm. However, this observation applies only to pressures of 150 and 200 MPa. Olsson [39] also notes the greater susceptibility to cracking of agglomerates produced from materials with lower degrees of comminution.

We found that the use of pressure caused a higher content of total polyphenols. In addition, the used pressure value also caused changes in the content of total polyphenols in agglomerated lemon balm samples. Cao et al. [40] reported that the content of phenolic compounds in strawberry pulp increased after using hydrostatic pressure. In addition, these authors reported that the value of pressure and the time of its application significantly affected the total phenolic content. Sadowska et al. [32] also reported that pepper mint agglomerates had a higher content of phenolic compounds compared to the non-agglomerated samples. On the basis of the results, a statistically significant effect of storage on the content of total polyphenols in lemon balm (*Melissa officinalis* L.) was demonstrated. A definitely higher content of total polyphenols (both fractions) was measured in samples directly after harvest than in samples stored for six months. Similar results were obtained by Jimenez-Zamora et al. [41], where a significant decrease in total polyphenols after six months of storage was observed, Kozłowska and Ścibisz [42] and Cao et al. [43]. In our study, we found that the content of total phenolic compounds decreased after 6 months of storage. This could be explained by the oxidation of phenolic compounds during the time of storage. In addition, enzymatic degradation could also reduce the content of phenolic compounds as well as the antioxidant activity. Similar results were reported by Sadowska et al. [32]. These authors found that the total polyphenol content of pepper mint agglomerates decreased by about 50% after three months of storage. According to data, total polyphenol content depends on many factors, such as climatic and cultivation conditions or harvest time. In studies carried out by Duda et al. [44], the highest amount of these compounds (6949–7643 mg·100 g^−1^) was determined in the extracts obtained from plants (only leaves) harvested at the maximum vegetative period. However, in Bulgarian lemon balm extracts much lower quantities of total polyphenols (1370 mg·100 g^−1^) were determined [45]. A very low content of total polyphenols (168.8 mg·100 g^−1^) was obtained by Sytar et al. [46]. It should be noted that the reported differences may also result from variations in the methods used for preparing research extracts [24]. In this study, the highest losses of total polyphenols were observed in the unpressed lemon balm herbs. We can suggest that the pressing of samples protects them against losing polyphenols, which is desirable. The highest content of total polyphenols was found in fraction 0.5–2.5 mm of lemon balm herbs pressed under 100 MPa among samples investigated directly after harvest. Considerably lower values were in the not-treated pressure samples in both fractions directly after harvest and after six months of storage. Confirmation of these relationships while using peppermint (*Mentha piperita*) can be found in the study by Sadowska et al. [32].

The results of antioxidant property studies of lemon balm (*Melissa officinalis*) measured using the ABTS test indicate a beneficial effect of pressure agglomeration. These findings are indirectly corroborated by research on the application of the modern method of high-pressure food preservation (HPP—High-Pressure Processing). In fruits and fruit-based products exposed to such pressure treatment, significantly higher antioxidant activity was observed compared to samples processed thermally [27,47]. As in the case of total polyphenols, the significantly lowest antioxidant activity was in the unpressed samples, in comparison with the other samples. This suggests that the pressing of lemon balm herb protects the antioxidant activity, both directly after obtaining the product and after a storage period. Similar observations for pressed peppermint (*Mentha piperita*) were made by Sadowska et al. [32]. Moreover, the literature lacks information on the effect of pressure agglomeration on the antioxidant properties of lemon balm (*Melissa officinalis*).

In the unpressed samples, the antioxidant activity directly after harvest in fraction 0.5–2.5 was 123.01 µmol Trolox∙g^−1^ and in fraction 2.5–5.0 was 117.29 µmol Trolox∙g^−1^. Lemon balm herb curves, which were obtained from the herb stored six months, slightly increased to 126.42 µmol Trolox∙g^−1^ in the first fraction and 131.73 µmol Trolox∙g^−1^ in the second fraction. Similar results to the results after six months were obtained by Dastmalchi et al. [48].

## 5. Conclusions

The results obtained in this study allow us to conclude that the agglomerate made from lemon balm (*Melissa officinalis* L.) exhibits satisfactory mechanical properties and high bioactive potential. Regardless of the plant fraction and applied compaction pressure, the mass loss of the agglomerate, determined in the abrasion test, was below 1% and met the pharmaceutical normative requirements for uncoated tablets. Considering the results of the mechanical tests, the recommended compaction pressure for this product is 200 MPa. Compaction of lemon balm, regardless of the fraction used and the pressure applied, resulted in an increase in the total polyphenol content and antioxidant properties, measured using the ABTS method, compared to the raw material that was not subjected to the agglomeration process, both immediately after preparation and after 6 months of storage. Thus, the achieved research results indicate a high utilitarian potential of lemon balm agglomerate.

## Figures and Tables

**Table 1:** Agglomerate resistance to abrasion, immediately after production.

Fraction (mm)	Pressure (MPa)	Agglomerate Abrasion Resistance (%)
Means of pressure	100	0.70 c *
	150	0.45 b
	200	0.38 a
Means for fraction		
0.5–2.5 mm		0.55 b
2.5–5.0 mm		0.46 a
Means for fraction and pressure		
0.5–2.5 mm	100	0.78 d
	150	0.48 b
	200	0.39 a
2.5–5.0 mm	100	0.62 c
	150	0.41 a
	200	0.39 a

* a, b, c, d—homogenous groups according to Duncan’s test *p* ≤ 0.05.

**Table 2:** Agglomerate resistance to abrasion after 3 months of storage.

Fraction (mm)	Pressure (MPa)	Agglomerate Abrasion Resistance (%)
Means of pressure	100	0.70 c *
	150	0.55 b
	200	0.47 a
Means for fraction		
0.5–2.5 mm		0.57 a
2.5–5.0 mm		0.58 a
Means for fraction and pressure		
0.5–2.5 mm	100	0.75 e
	150	0.52 bc
	200	0.44 a
2.5–5.0 mm	100	0.64 d
	150	0.59 cd
	200	0.50 ab

* a, b, c, d, e—homogenous groups according to Duncan’s test *p* ≤ 0.05.

**Table 3:** Mechanical properties of the agglomerate in the Brazilian test.

Fraction (mm)	Pressure (MPa)	Brazilian Test Results (MPa)	Agglomerate Diameter (mm)	Agglomerate Height (mm)
Means of pressure	100	1.352 a *	15.909	9.877
	150	1.773 b	15.872	9.288
	200	1.958 c	15.847	9.014
Means for fraction				
0.5–2.5 mm		1.724 b	15.860	9.360
2.5–5.0 mm		1.665 a	15.892	9.424
Means for fraction and pressure				
0.5–2.5 mm	100	1.340 a	15.901	9.899
	150	1.819 c	15.845	9.264
	200	2.013 e	15.835	8.917
2.5–5.0 mm	100	1.364 a	15.920	9.850
	150	1.727 b	15.898	9.311
	200	1.903 d	15.858	9.111

* a, b, c, d, e—homogenous groups according to Duncan’s test *p* ≤ 0.05.

**Table 4:** Bulk density of agglomerate.

Fraction (mm)	Pressure (MPa)	Agglomerate Density (kg·m^−3^)
Means of pressure	100	1019.306 a *
	150	1090.629 b
	200	1125.520 c
Means for fraction		
0.5–2.5 mm		1086.458 b
2.5–5.0 mm		1070.511 a
Means for fraction and pressure		
0.5–2.5 mm	100	1018.891 a
	150	1099.198 c
	200	1141.285 e
2.5–5.0 mm	100	1019.721 a
	150	1082.059 b
	200	1109.755 d

* a, b, c, d, e—homogenous groups according to Duncan’s test *p* ≤ 0.05.

**Table 5:** Total polyphenol concentration and antioxidant activity of lemon balm directly after compaction process.

Fraction (mm)	Pressure (MPa)	Total Polyphenols ± SD (mg·100 g^−1^)	ABTS^+^ ± SD (μmol Trolox·g^−1^)
	0	2017.39 ± 166.07 a *	120.15 ± 3.82 A **
Means of pressure	100	3357.37 ± 439.10 d	154.83 ± 10.26 B
	150	3045.19 ± 266.67 c	155.04 ± 2.80 B
	200	2762.48 ± 507.04 b	167.96 ± 18.72 C
Means for fraction			
0.5–2.5 mm		3108.59 ± 611.21 b	152.61 ± 19.43 B
2.5–5.0 mm		2482.63 ± 445.62 a	146.38 ± 22.31 A
Means for fraction and pressure			
	0	2156.76 ± 85.60 b	123.01 ± 1.62 A
0.5–2.5 mm	100	3762.81 ± 70.98 g	160.67 ± 1.20 C
	150	3283.96 ± 108.43 f	156.70 ± 3.17 C
	200	3230.81 ± 64.43 f	170.06 ± 13.77 D
	0	1878.03 ± 72.33 a	117.29 ± 3.09 A
2.5–5.0 mm	100	2951.92 ± 80.56 e	175.25 ± 10.13 D
	150	2806.42 ± 46.29 d	153.38 ± 0.96 C
	200	2294.14 ± 104.06 c	139.61 ± 3.11 B

* a, b, c, d, e, f, g—homogenous groups for total polyphenol content according to Duncan’s test *p* ≤ 0.05. ** A, B, C, D—homogenous groups for antioxidant properties determined by the ABTS method according to Duncan’s test *p* ≤ 0.05.

**Table 6:** Total polyphenol concentration and antioxidant activity of lemon balm after six months of storage.

Fraction (mm)	Pressure (MPa)	Total Polyphenols ± SD (mg·100 g^−1^)	ABTS^+^ ± SD (μmol Trolox·g^−1^)
	0	630.53 ± 167.18 a *	129.08 ± 6.96 A **
Means of pressure	100	1524.55 ± 97.12 c	161.17 ± 8.65 B
	150	1483.73 ± 104.49 c	162.33 ± 5.76 B
	200	1328.59 ± 83.23 b	161.20 ± 9.50 B
Means for fraction			
0.5–2.5 mm		1296.57 ± 290.44 a	150.49 ± 15.69 A
2.5–5.0 mm		1214.53 ± 449.97 a	156.40 ± 16.52 B
Means for fraction and pressure			
	0	769.08 ± 110.98 b	126.42 ± 7.93 A
0.5–2.5 mm	100	1456.80 ± 52.73 de	155.13 ± 1.12 A
	150	1472.27 ± 135.12 def	162.45 ± 8.51 A
	200	1365.71 ± 85.50 cd	157.94 ± 5.35 A
	0	491.98 ± 41.32 a	131.73 ± 5.61 A
2.5–5.0 mm	100	1592.30 ± 83.59 f	167.22 ± 8.71 A
	150	1500.93 ± 40.79 ef	162.20 ± 2.27 A
	200	1272.90 ± 41.32 c	164.46 ± 12.39 A

* a, b, c, d, e, f —homogenous groups for total polyphenol content according to Duncan’s test *p* ≤ 0.05. ** A, B—homogenous groups for antioxidant properties determined by the ABTS method according to Duncan’s test *p* ≤ 0.05.

## Data Availability

The original contributions presented in the study are included in the article, further inquiries can be directed to the corresponding author.
